# Continuous centrifugal microfluidics (CCM) isolates heterogeneous circulating tumor cells via full automation

**DOI:** 10.7150/thno.72511

**Published:** 2022-05-01

**Authors:** Hyeong Jung Woo, Seung-Hoon Kim, Hyo Jung Kang, Soo-Hwan Lee, Seung Joon Lee, Jong Man Kim, Ogan Gurel, Soo Yeol Kim, Hye Ran Roh, Jungmin Lee, Yeonsoo Park, Hyun Young Shin, Yong-Il Shin, Sun Min Lee, So Yeon Oh, Young Zoon Kim, Jung-Il Chae, Seoyoung Lee, Min Hee Hong, Byoung Chul Cho, Eun Sook Lee, Klaus Pantel, Hye Ryun Kim, Minseok S. Kim

**Affiliations:** 1 Department of New Biology, DGIST, Daegu 42988, Korea.; 2 CTCELLS Inc., 206-C, R7, DGIST, Daegu 42988, Korea.; 3 JE-UK Institute for Cancer Research, JEUK Co. Ltd., Gumi-si 39418, Korea.; 4 College of Transdisciplinary Studies, DGIST, Daegu 42988, Korea.; 5 Research Institute for Convergence of Biomedical Science and Technology, Pusan National University Yangsan Hospital, Yangsan 50612, Korea.; 6 Department of Rehabilitation Medicine, Pusan National University School of Medicine, Yangsan 50612, Korea.; 7 Department of Laboratory Medicine, Pusan National University Yangsan Hospital, Pusan National University School of Medicine, Yangsan 50612, Korea.; 8 Medical Oncology & Hematology, Department of Internal Medicine, Pusan National University Yangsan Hospital, Yangsan 50612, Korea.; 9 Division of Neurooncology and Department of Neurosurgery, Samsung Changwon Hospital, Sungkyunkwan University School of Medicine, Changwon 51353, Korea.; 10 Department of Dental Pharmacology, School of Dentistry, Jeonbuk National University, Jeonju 54896, Korea.; 11 Division of Medical Oncology, Department of Internal Medicine, Yonsei University College of Medicine, Gangnam Severance Hospital, Seoul 06273, Korea.; 12 Division of Medical Oncology, Department of Internal Medicine, Yonsei Cancer Center, Yonsei University College of Medicine, Seoul 03722, Korea.; 13 Center for Breast Cancer, National Cancer Center, Goyang 10408, Korea.; 14 Institute of Tumour Biology, University Medical Centre Hamburg-Eppendorf, Hamburg 20246, Germany.

**Keywords:** cancer heterogeneity, circulating tumor cells, continuous centrifugal microfluidics, unbiased isolation, full automation

## Abstract

Understanding cancer heterogeneity is essential to finding diverse genetic mutations in metastatic cancers. Thus, it is critical to isolate all types of CTCs to identify accurate cancer information from patients. Moreover, full automation robustly capturing the full spectrum of CTCs is an urgent need for CTC diagnosis to be routine clinical practice.

**Methods:** Here we report the full capture of heterogeneous CTC populations using fully automated, negative depletion-based continuous centrifugal microfluidics (CCM).

**Results:** The CCM system demonstrated high performance (recovery rates exceeding 90% and WBC depletion rate of 99.9%) across a wide range of phenotypes (EpCAM(+), EpCAM(-), small-, large-sized, and cluster) and cancers (lung, breast, and bladder). Applied in 30 lung adenocarcinoma patients harboring epidermal growth factor receptor (EGFR) mutations, the system isolated diverse phenotypes of CTCs in marker expression and size, implying the importance of unbiased isolation. Genetic analyses of intra-patient samples comparing cell-free DNA with CCM-isolated CTCs yielded perfect concordance, and CTC enumeration using our technique was correlated with clinical progression as well as response to EGFR inhibitors.

**Conclusion:** Our system also introduces technical advances which assure rapid, reliable, and reproducible results, thus enabling a more comprehensive application of robust CTC analysis in clinical practice.

## Introduction

**Figure 1:**
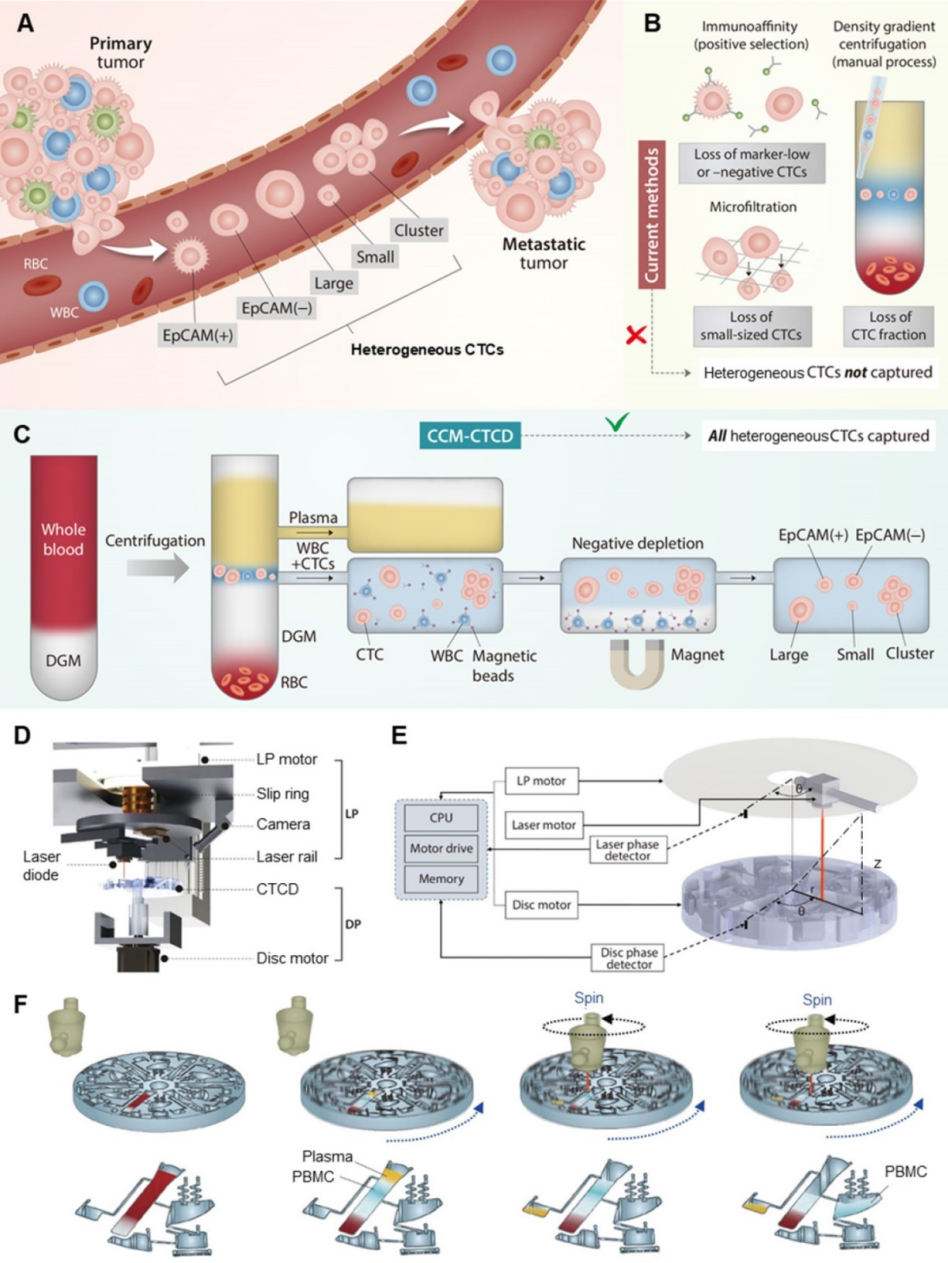
** CTC extraction strategy and operation of CCM-CTCD system. (A)** CTCs, which arise from primary tumors and may give rise to metastases, manifest heterogeneous (polyphyletic) phenotypes. **(B)** Current methods of CTC isolation, which include marker-dependent (immunoaffinity/positive selection) as well as marker-independent (microfiltration and density gradient centrifugation) techniques, fail to fully capture heterogeneous CTCs. **(C)** Our CCM-CTCD extraction strategy resulting in the capture of all polyphyletic CTCs. **(D)** CCM-CTCD system components. LP and DP denote the laser part and disc part, respectively. **(E)** CCM-CTCD system is fully automated with the laser and disc rotations being synchronized by reference to the radius and phase (angular coordinates) of a specified point on the disc. **(F)** Schematic of CCM-CTCD operation to precisely extract the PBMC layer. Because the laser module rotates synchronously with the disc, plasma and PBMC layers are precisely moved to respective chambers under maintaining centrifugal force.

Cancer cell heterogeneity is a critical characteristic in cancer biology and clinical oncology, contributing to cancer progression, metastasis, neoplastic drug resistance, and relapse 1, 2. Blood-borne dissemination of tumor cells and outgrowth in secondary organs are the hallmarks of cancer metastasis as a leading cause of cancer-related death. Thus, blood analysis, also designated as “liquid biopsy”, offers unique insights into tumor evolution in individual cancer patients 3. As circulating tumor cells (CTCs) present in the blood circulation also manifest significant heterogeneity (Figure 1A), the isolation and analysis of such varied CTC populations have been still challenging 4, 5. The phenotype-specific separation strategies used by conventional CTC isolation techniques are inherently limited in overcoming CTC heterogeneity. While these methods may report high recovery rates, cells not targeted by the isolation principle will necessarily be missed. Undercounting of heterogeneous cells calls into question CTC enumeration as a reliable clinical biomarker, thus representing a significant gap in the technology 5. Therefore, it is necessary to capture the full spectrum of CTCs 6. This requires an unbiased isolation technique with high recovery rates regardless of cancer cell characteristics. Additionally, full automation is another essential requirement for CTC analysis to be a routine clinical practice.

There are two approaches, marker-dependent and marker-independent, to distinguishing and separating CTCs from plasma, red blood cells (RBCs) and white blood cells (WBCs) (Figure 1B) 7. In the first, marker-dependent methods use antibodies of CTC surface markers such as epithelial cell adhesion molecule (EpCAM). Such antibodies are conjugated to physically separable magnetic nanoparticles, microposts, or microfluidic surfaces, thus enriching the CTCs via positive selection 7, 8. These techniques are difficult to isolate CTCs expressing little or no marker 9, 10. In the second approach, CTCs are marker-independently distinguished via physical parameters of cells such as size or density 11. As a label-free isolation approach, many types of principles including microfiltration 12, acoustic 13, deterministic lateral displacement (DLD) 14, spiral 15, electric 3, magnetic 16, optical 17, and inertial 18 separation technologies have been introduced. While the technologies have individual key features with their physical mechanisms, the final separation criteria for the technologies are based on size. Since their technologies are based on the assumption that CTCs are relatively larger than WBCs, they can easily miss small-sized CTCs and be limited in purity by relatively large leukocytes and their clogging 19. As another label-free isolation approach, density-based isolation using density gradient media (DGM) is used to separate particular cells in blood 20, 21. Since the methods are usually conducted with manual pipetting of the gradient layer, they are imprecise, labor-intensive, operator-dependent, and often resulting in incomplete CTC extraction and WBC contamination 4. To overcome the challenges, OncoQuick^®^ 22, 23, RosetteSep^TM^ 24, RareCyte^®^ 25, Dynabead 26, and other modified studies based on density have been introduced 22. While they have a critical advantage with marker- and size-independent isolation, they still showed relatively low purity 27 and recovery rate because of the entrapment of CTCs in the leukocyte-RBC complexes 28 as well as manual or partial automation, which makes them difficult as a routine clinical setting. To summarize: as few reports have been introduced for both marker- and size-independent CTC isolation with full automation 29, 30, a fully-automated unbiased method to capture diverse CTC phenotypes is still lacking 30.

This report outlines the design, optimization, evaluation, and clinical application of Continuous Centrifugal Microfluidics - Circulating Tumor Cell Disc (CCM-CTCD), in which fully automated microfluidic extractions are performed during continuous centrifugation combined with WBC negative depletion, resulting in the unbiased capture of diverse and viable CTC populations (Figure 1C). A precise extraction of ultra-thin fluid layers of CCM-CTCD during a rotational state permits high recovery rates and purity (recovery rates exceeding 90% and depletion rate of 99.9%) of a wide range of CTC subtypes including EpCAM(+), EpCAM(-), small and large cancer cells along with CTC clusters. In addition, when applied in a clinical validation study with 30 lung adenocarcinoma patients harboring epidermal growth factor receptor (EGFR) mutations, the system successfully isolated the full spectrum of CTCs. The isolated CTCs from patients' blood showed heterogeneity in their EpCAM expression and sizes, which were relatively small (median diameter, less than 9 μm). These heterogeneities indicate that marker-based or size-based isolation might fail to isolate marker negative or small CTCs and miss important oncology information of patients. Genetic analyses of intra-patient samples comparing cell-free DNA (cfDNA) with CCM-isolated CTCs yielded perfect concordance. They were correlated with clinical progression as well as response to EGFR-targeted therapy.

## Results

### CCM-CTCD precisely extracts the ultra-thin PBMC layer

A common method for active control in centrifugal microfluidics operates via laser-irradiation of ferrowax microvalve 31. With current techniques, moving the laser to a specific valve requires the rotation to stop, causing mixing of the separated layers, imprecise extraction, and consequent loss of CTC fractions and WBC contamination (Figure S1). With CCM-CTCD, synchronous rotation of the system's disc part (DP) and laser part (LP) permits microfluidic operations during continuous centrifugation (Figure 1D). Once the DP commences spinning, and after a certain optimized duration of time, the laser motor begins rotating with its angular velocity and phase becoming matched to that of the rotating disc based on phase values provided by disc and laser phase detectors (Figure 1E). Once the DP and LP rotations are phase-locked, the laser diode moves to the corresponding wax valve, the opening of which causes microfluidic transfers to the succeeding chamber. During these microfluidic transfers, the disc continuously rotates, maintaining thin layer geometries and thus enabling precise extraction (Figure 1F).

### Operation and optimization of CCM-CTCD

**Figure 2:**
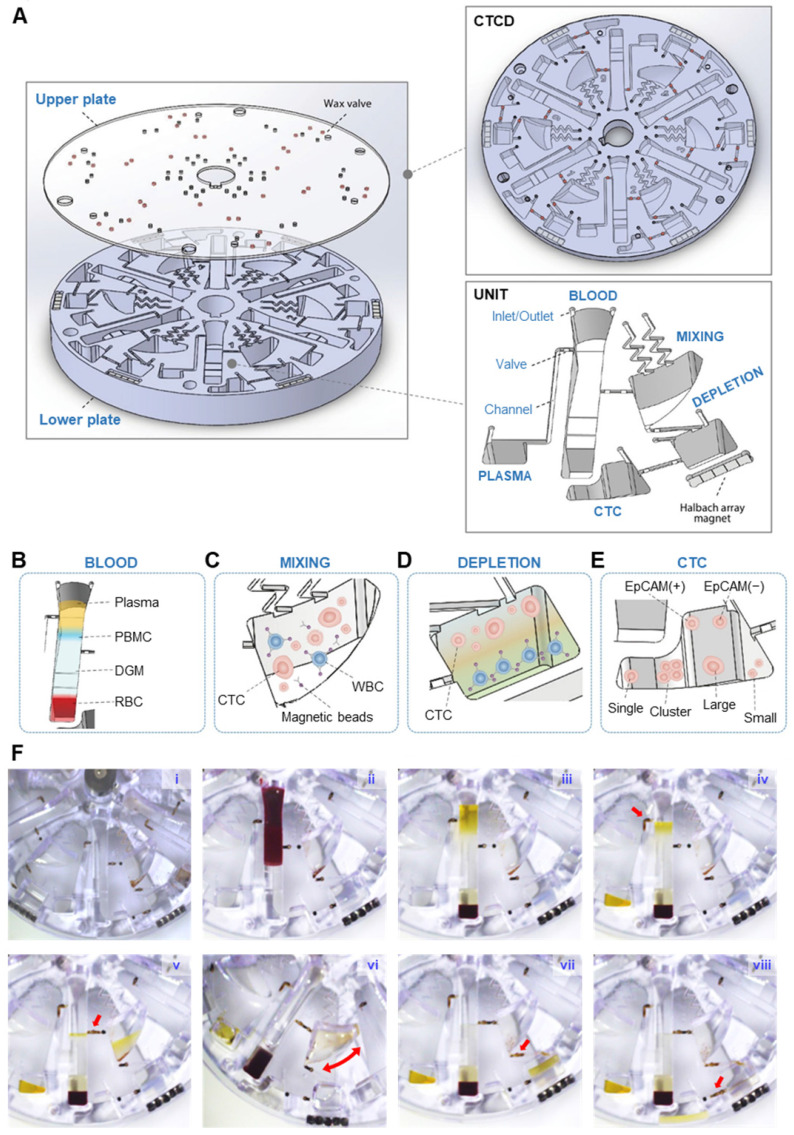
** Isolation process of the CTCs using CTCD. (A)** Structure of CTCD. The disc is constructed from an upper plate, which includes the wax valves, bonded to a lower plate. **(B-E)** CCM-CTCD chamber architectures showing successive enrichment of CTCs starting from the BLOOD chamber **(B)** ultimately reaching the CTC chamber. The PBMC layer is transferred from the BLOOD chamber to the MIXING chamber **(C)** wherein the WBCs are bound to anti-CD45 conjugated magnetic microbeads. **(D)** These WBC-microbead complexes then sink to the bottom of the DEPLETION chamber and **(E)** only CTCs floating above the DGM are transferred to the CTC chamber. **(F)** Successive images of CCM-CTCD enrichment and isolation process. **(F-i)** Image of the disc with DGM (at the bottom) in the BLOOD chamber and prior to loading of the patient's blood sample. **(F-ii)** Injection of the whole blood sample into the BLOOD chamber. **(F-iii)** Centrifugal process to form PBMC. **(F-iv)** Removing plasma layer. The red arrow shows the valve opening to remove the plasma layer via the microchannel leading to the PLASMA chamber. **(F-v)** Transfer the PBMC layer to the MIXING chamber. The red arrow indicates the valve opening to transfer the PBMC layer. **(F-vi)** Shaking process for bead binding in the MIXING chamber. **(F-vii)** Centrifugation to deplete WBCs in the DEPLETION chamber. The red arrow denotes the transfer of the mixture of CTCs and microbead-bound WBCs to the DEPLETION chamber. **(F-viii)** Extraction of CTCs. By opening the valve (red arrow), CTCs are moved to the final CTC chamber.

The CTCD comprises upper and lower plates with six identical isolation units. Each unit consists of five chambers - PLASMA, BLOOD, MIXING, DEPLETION, and CTC - along with microfluidic components such as inlet/outlet ports, valves, channels, and Halbach array magnet (Figure 2A). CTCs are captured through two successive spins, interposed with a mixing stage. The peripheral blood mononuclear cells (PBMCs) are first separated via centrifugation from plasma and RBCs (Figure 2B). The extracted PBMCs then undergo mixing with anti-CD45 conjugated magnetic microbeads (Figure 2C), followed by second centrifugation that separates, via negative depletion, the WBCs (Figure 2D) from CTCs (Figure 2E). The process is described in more detail in Table S1 and Movie S1.

Figure 2F and Movie S2 show the step-by-step operation to isolate heterogeneous CTCs. First, DGM is layered at the bottom of the BLOOD chamber (Figure 2F-i), into which whole blood is injected (Figure 2F-ii). This chamber's contents are then separated via centrifugation into four layers with the plasma and PBMC layers being moved (Figure 2F-iii) to the PLASMA (Figure 2F-iv) and MIXING chambers (Figure 2F-v), respectively. Centrifugation is paused and the MIXING chamber, pre-filled with anti-CD45 conjugated magnetic microbeads, and now containing WBCs and CTCs, undergoes shaking (Figure 2F-vi). Following shaking, centrifugation resumes and the CTCs and microbead-bound WBCs flow under microfluidic control into the DEPLETION chamber (Figure 2F-vii). The denser microbead-conjugated WBCs become separated from the CTCs, allowing the enriched CTCs to flow into the final CTC chamber (Figure 2F-viii). CCM-CTCD was superior to conventional (manual) centrifugation methods in terms of WBC depletion and CTC recovery rates.

**Figure 3:**
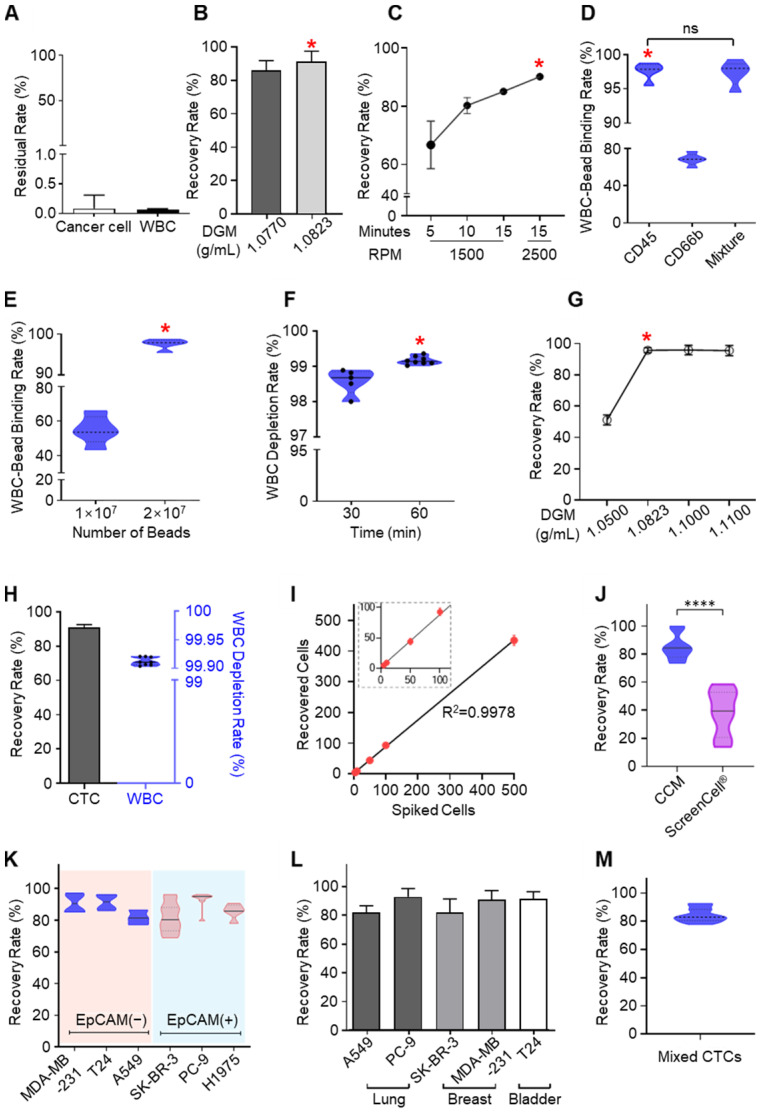
** Optimization and performance of CCM-CTCD. (A)** Cancer cells and WBCs remaining in the PLASMA chamber were negligible. For CTCs this residual rate was less than 0.09% while the corresponding rate for WBCs was less than 0.07%. **(B)** The recovery rate depending on the DGM density in the BLOOD chamber. **(C)** Recovery rate as a function of RPM and centrifugation time in the BLOOD chamber. **(D)** Binding rate of microbeads for WBCs as a function of different antibodies in the MIXING chamber. **(E)** Binding rate as a function of microbead numbers injected into the MIXING chamber. When 2 × 10^7^ microbeads were injected, the binding rate reached 99%. **(F)** Depletion rate depending on the bead binding time. **(G)** CTC recovery rate according to DGM density in the DEPLETION chamber. **(H)** Final recovery rate and WBC depletion rate. The final result, combining all optimized parameters, yielded a 92% recovery rate and 99.9% depletion rate. **(I)** Regression analysis of recovered cells versus spiked cell numbers. The CCM platform showed highly reliable and robust performance. **(J)** The recovery rate for H1688, a small cell lung cancer cell line (< 10 µm). As compared to the microfiltration approach (ScreenCell^®^), CCM-CTCD showed a significantly higher recovery rate for these small cells (****p < 0.0001). **(K)** Recovery rate as a function of cancer cell EpCAM expression. The CCM-CTCD technique achieved high recovery rates regardless of EpCAM expression status. **(L)** Recovery rate as a function of cancer types. Again, CCM-CTCD resulted in similar, reproducible recovery rates across different cancer types. **(M)** The recovery rate for mixed cancer cells (20 cells per A549, PC-9, SK-BR-3, MDA-MB-231, and T24 cell lines) with different phenotypes. In **B-G**, the red asterisk represents the optimal condition for each of the respective experiments.

We next evaluated and optimized the system's operational parameters (Figure 3A-H). Residual CTCs in the plasma extract were less than 0.1% of the total CTC number, attributable to the precise extractions enabled by continuous centrifugation (Figure 3A) 32, 33. For the BLOOD and DEPLETION chambers, optimal DGM density was 1.0823 g/mL (Figure 3B and 3G), a rotation rate of 2500 RPM, and a spin time of 15 minutes also resulted in maximal recovery rates (Figure 3C). We optimized the MIXING chamber shaking conditions to be at an angle and frequency of 135° and 1 Hz, respectively, the ideal WBC surface marker to be anti-CD45 conjugated microbeads (Figure 3D), and 2×10^7^ immunomagnetic beads (Figure 3E) with a binding time of 1 hour (Figure 3F). With all such parameters optimized, the CCM-CTCD platform showed a 92% recovery rate and 99.9% depletion rate (Figure 3H). The CTC recovery rate was reproducible across a wide range of initial spiked numbers, from a few to hundreds (Figure 3I).

### CCM-CTCD captures heterogeneous CTCs

Current CTC isolation methods report a wide range of recovery rates from near 0% up to 87% in the identical method 26, 27. As recovery rates vary by CTC cell type, a median recovery rate is often cited. Parsortix system, for example, reports a CAKI-2 cell recovery rate as high as 87%, but the 66% median rate implicitly acknowledges that heterogeneous CTCs cannot be fully captured 26. Some EpCAM-based positive selection systems, while effective for that phenotype, have nonetheless shown recovery rates of less than 30% 26. Our group has also explored different CTC isolation platforms 19, 31, 34, 35. Still, the recovery rates similarly depended on cancer cell phenotype, and we were unable to achieve a generally high recovery rate for heterogeneous CTC populations.

With the CCM-CTCD, we have successfully captured a wide range of CTC types. Compared to an established filter-based approach (ScreenCell^®^), which recovered 39% of small CTCs, our system exhibited a superior and reproducible 90% recovery rate (Figure 3J). With EpCAM(+) and EpCAM(-) CTCs, we obtained reproducibly high recovery rates ranging between 82% - 93% for both cell types (Figure 3K). The CCM-CTCD also reliably showed high recovery rates (mean of 87.7%) across a range of cancers, including lung (A549, PC9, and H1975), breast (MDA-MB-231, and SK-BR-3), and bladder (T24) cancer cell lines (Figure 3L). To model a multi-metastatic disease, five cancer cell lines were mixed with different phenotypes (EpCAM(+), EpCAM(-), small-sized, and large-sized CTCs) and the recovery rate was confirmed to emphasize the usefulness of CCM-CTCD even in multi-metastatic cancer patients. As expected, the result showed a high recovery rate (84.3%), reconfirming the isolation performance unlimited to CTC heterogeneity (Figure 3M).

To the best of our knowledge, the CCM-CTCD system represents the first demonstration of a CTC mixture model with various phenotypic subtypes, achieving a high recovery rate. Overcoming heterogeneity is also critical in two other circumstances. First, in demonstrating CCM-CTCD's capability across a range of cancer lines, this technique may be helpful to multi-metastatic cancer patients. Second, while some current CTC isolation techniques show promise for particular cancers, CCM-CTCD, with its generality, suggests the possibility of being a general platform across all cancers.

### Clinical validation of CCM-CTCD

Applying our systems to clinical practice, we wanted to answer several questions. First, we sought to determine if we could isolate heterogeneous CTCs from intra-patient samples. Second was the question of whether we could acquire mutational information. Third, we wanted to investigate if CTC enumeration would correlate with clinical progression. Fourth, we needed to determine if this information could be optimized for targeted therapy to overcome resistant mutants. We used the CCM-CTCD to study 30 non-small cell lung cancer (NSCLC) patients, with documented EGFR mutations in their tumor tissues. Our results provided affirmative answers to these questions.

**Figure 4:**
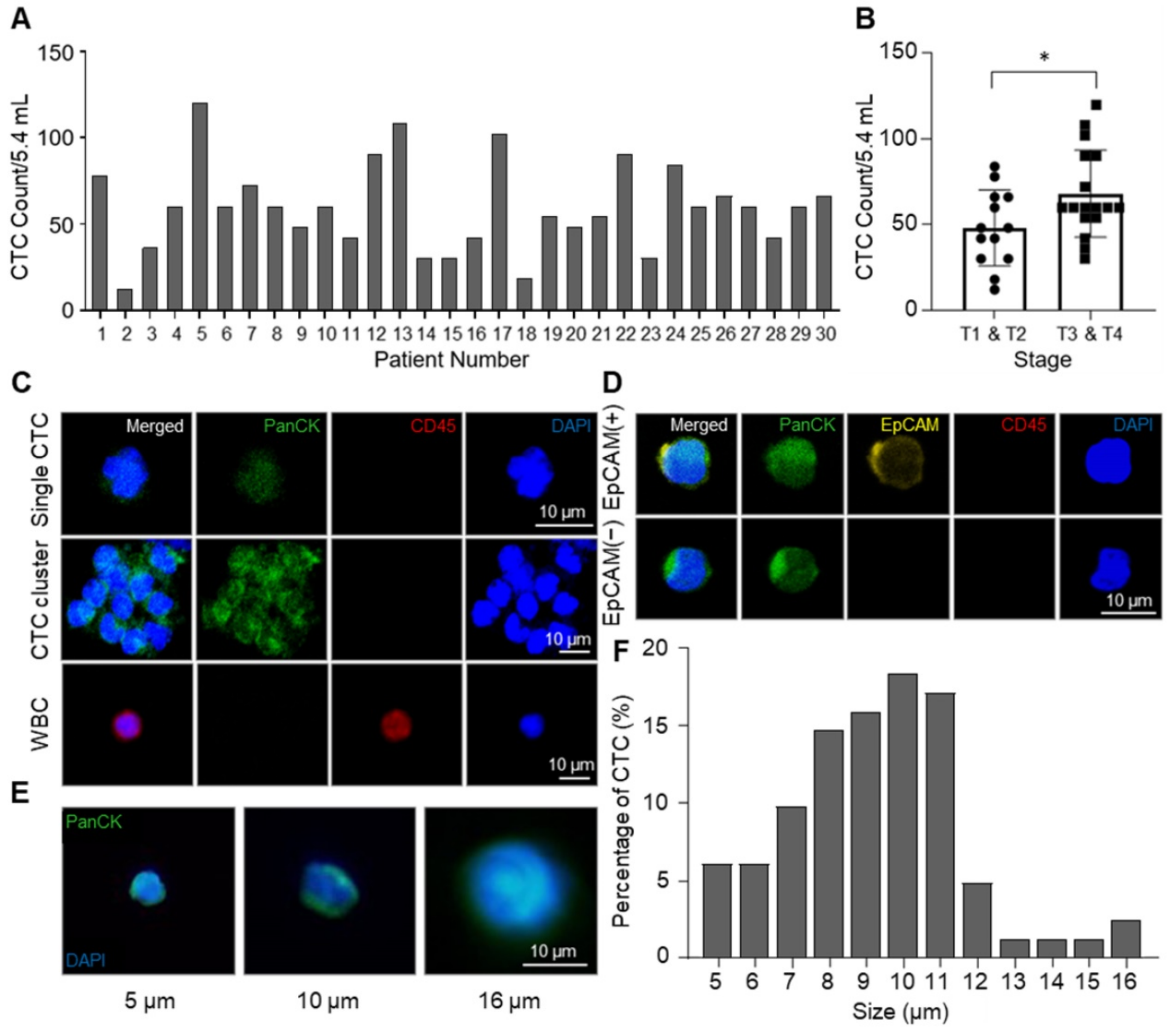
** Enumeration and identification of CTC with high heterogeneity. (A)** Total numbers of isolated CTCs in 5.4 mL of blood. Bar plot showing the number of CTCs counted from NSCLC patients' blood samples using CCM-CTCD. **(B)** Correlation between CTCs number and cancer stages (*p < 0.05). **(C)** Immunofluorescence images of CTCs isolated from lung cancer patients. The top row shows a single CTC with DAPI(+)/PanCK(+)/CD45(-); middle row shows a CTC cluster; bottom row shows a WBC with DAPI(+)/PanCK(-)/CD45(+). **(D)** Fluorescence images of the isolated CTCs for EpCAM-positive and -negative cells. **(E)** Representative images showing a variety of CTC sizes captured including 5 (left), 10 (middle), and 16 (right) µm sized CTCs. **(F)** Histogram plot of the percentage of CTCs as a function of size (µm). Based on these results, the CCM-CTCD platform in this clinical setting showed full capture of diverse CTC phenotypes (single, clustered, EpCAM(+), EpCAM(-), small-sized, and large-sized CTCs).

From NSCLC patients, CTCs were isolated by the CCM-CTCD and identified by pan-cytokeratin (PanCK) staining. The CCM-CTCD successfully isolated an average of 59.4 CTCs from 5.4 mL of NSCLC patients' blood ranging from 12 to 120 cells (Figure 4A). In general, we observed significantly higher CTC counts in the patients of later stages (T3, T4) when compared to those in earlier stages (T1, T2). However, CTC counts correlated with neither the regional lymph nodes metastasis stages nor distant metastasis stages (Figure 4B and S2). Compared to size-based isolation and EpCAM-based isolation methods that detected about 20 CTCs as a median value from 7.5 mL of NSCLC patient blood 36-38, CCM-CTCD detected 60 CTCs as a median value from 5.4 mL of blood, almost 4-fold more CTCs, which could be the results that CCM-CTCD isolated various subtypes of CTCs. In addition, considering the result that CTCs were isolated from all patients, even in early stages, it is shown that CCM-CTCD has high sensitivity and suggests that it has a potential to be used for early-stage diagnosis. Specifically, we showed that CCM-CTCD could isolate the full spectrum of CTC types (EpCAM(+) and EpCAM(-), small- and large-sized CTCs including clusters) found in NSCLC (Figure 4C-E). The size distribution of CTCs showed considerable heterogeneity ranging from 5 μm to 16 μm, with a majority (71%) found to be small-sized (< 10 μm) (Figure 4F). Similar to the previous studies in which 4 ~ 6 μm sized CTCs were found 16, 39, the CCM-CTCD was also able to detect multiple small-sized CTCs. Thus, the size distribution of CTCs indicated size-based isolation might miss a large portion of CTCs.

**Figure 5:**
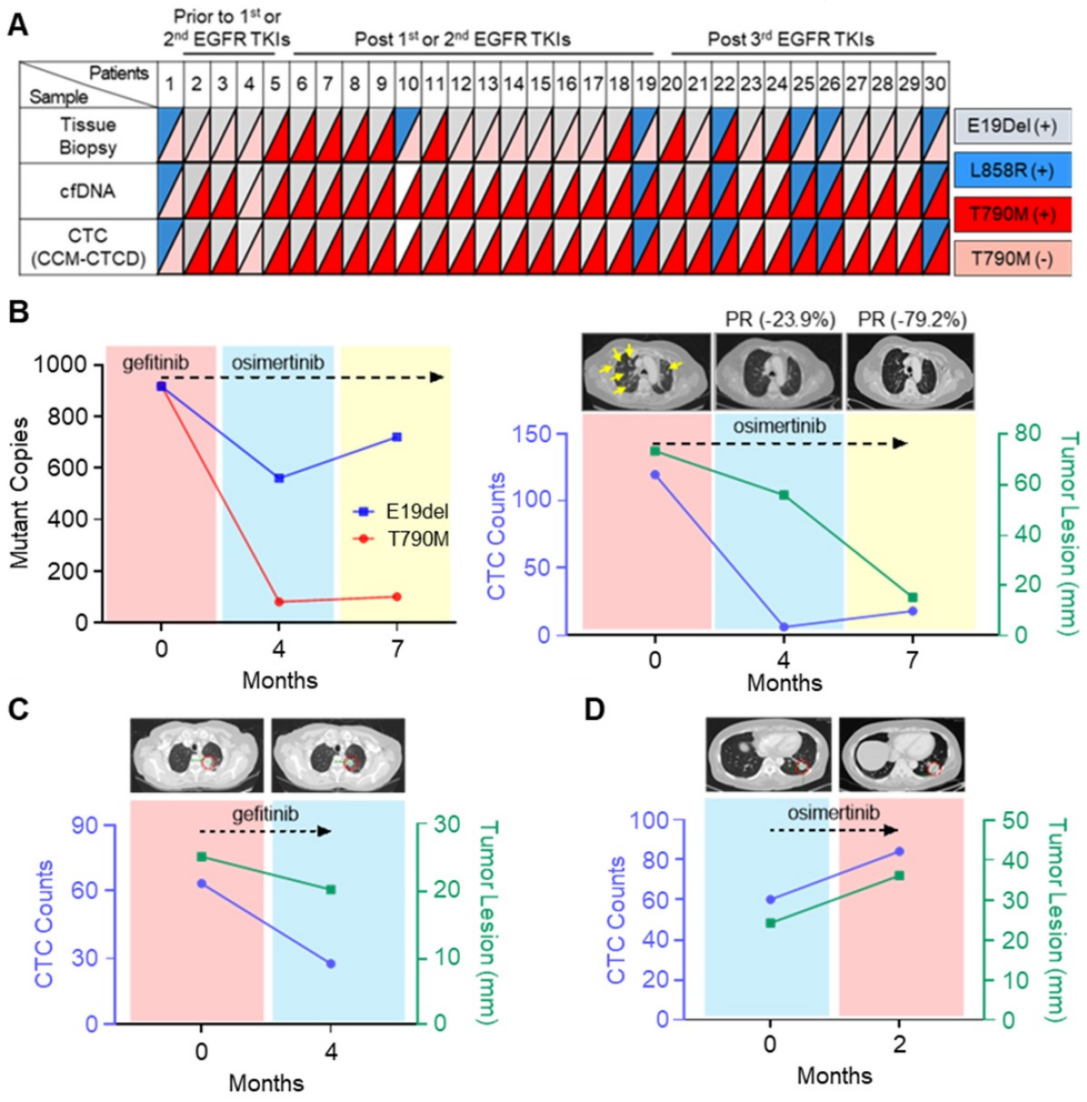
** Mutational analysis and longitudinal follow-up of CTC samples from NSCLC patients. (A)** EGFR mutation profiles of matched tissue biopsy, cfDNA, and CTCs of 30 lung cancer patients. A perfect agreement was shown between CTCs and cfDNA while a slight agreement was seen between CTCs and tissue biopsy.** (B)** Longitudinal follow-up of mutant copies with CTC-derived DNA (left panel) and correlation between tumor lesion and CTC number (right panel) in patient #5. EGFR mutant copy number decreased from baseline after optimized EGFR-TKI treatment. In addition, radiographic evidence of regression of metastases (yellow arrows) was also correlated with decreased CTC counts from the onset of treatment. **(C)** CT scan of patient #4's chest shows the primary lesion (within red circle). Lung images prior to treatment of osimertinib and at progression showing that during the seven-month osimertinib treatment, the primary lesion significantly diminished in size. Serial CTC counts were indicated at each time point. **(D)** Longitudinal follow-up of changes for CTC numbers of patient #27, with progressive disease. In this case, the primary lesion (circled in red) has increased in size, as the CTC counts have increased. CTC counts represent the CTC number per 5.4 mL of peripheral blood.

From the isolated CTCs, EGFR mutations were identified by droplet digital PCR (ddPCR) and pyrosequencing (Table S2). Intra-patient EGFR mutations identified in the isolated CTCs were perfectly concordant with the mutations found in tumor-derived cfDNA, but not in tissue biopsy (Figure 5A and Table S3). In some cases, there are mismatches between CTC and tissue results originating from the temporal gap. Since the patients marked with asterisks in Table S3 are very difficult to try re-biopsy due to the patient's poor general condition, there is at least a year gap between tissue and CTC analysis. However, discrepancies also occurred even when the time gap between the tissue and the CTC analysis was not significant. Previous studies have shown that these mismatches sometimes happen due to the different mutation analysis methods between tissues and CTCs, or the tissue biopsy does not reflect the tumor heterogeneity 40. Patient #5, who experienced abrupt disease progression during gefitinib treatment, a first-generation EGFR-tyrosine kinase inhibitor (TKI), offered an instructive example. Using CCM-CTCD, we isolated the patient's CTCs and found both E19del and T790M mutations conferring treatment resistance, which matched that observed with the patient's blood cfDNA. We started osimertinib, the 3rd generation EGFR-TKI standard therapy for T790M+ patients, after four months, leading to a significant reduction in the mutant copy number (Figure 5B). Likewise, the decreased CTC counts correlated with the clinical response as observed with the near disappearance of multiple lung metastases on computerized tomography (CT) (yellow arrows of Figure 5B). In another example (patient #4), we observed a CTC count decrease following gefitinib treatment, which strongly correlated with partial clinical response (Figure 5C). In another patient (#27), CTC counts abruptly increased after osimertinib, in line with clinical progression based on CT (Figure 5D). Collectively, these results indicated that CCM-CTCD successfully isolates genuine CTCs from EGFR mutant NSCLC patients. Our system isolates the full spectrum of phenotypically distinct CTCs and we can identify specific EGFR mutants in these intra-patient isolated CTC. Therefore, such information can be used to optimize targeted therapy to overcome drug-resistant variants. Finally, we validated that CTC enumeration is correlated with both clinical monitoring and therapeutic response.

## Discussion

While many outstanding pioneering efforts in CTC technology have promised to significantly impact cancer care 14, 15, 41, 42, clinical adoption of this technology has been limited. We speculate the major reason for this disconnect between promise and reality: most separation approaches are difficult to simultaneously satisfy overcoming cancer cell heterogeneity and practical use in a clinical setting. This report describes CCM-CTCD, a CTC enrichment and isolation technology that directly overcomes the challenge. The current gold standard for CTC isolation is the FDA-approved CellSearch^®^ system, which uses EpCAM antibody-based magnetic cell sorting - again, likely to miss EpCAM(-) CTCs 8, 9. Since then, there have been many efforts to capture heterogeneous CTC populations 18, 43-53. One promising approach uses an integrated microfluidic system that combines hydrodynamic and magnetic cell sorting 18, 43, 44. However, these methods require additional preprocessing of blood before isolating CTC such as RBC lysis or antibody reaction 18, 43, 44. Thus, full automation might not be easy. Other CTC isolation techniques utilize magnetic sorting 45, 47, marker-based isolation 46, filter based isolation 48-50, and spiral microfluidics 51-53. While these techniques offer several advantages including automation, they are fundamentally size-based separation 48-53 or marker-based separation 45-47. Therefore, there are few reports yet to achieve full automation while realizing the isolation of heterogeneous CTCs. Since CTC enumeration as a biomarker via liquid biopsy is only valid if the technique is impartial and reproducible, an unbiased isolation technology is essential for accurate clinical treatment selection and prognosis. Incomplete enumeration, either because of polyphyletic CTCs being missed or through drug-mediated removal of specific populations, is unlikely to be of clinical value. In this regard, the CCM-CTCD system introduced in this study can be a valuable tool to improve the diagnostic credibility of CTCs. Via the novel innovation of continuous centrifugation, the CCM-CTCD transformed the traditional density-based isolation methods into a powerful, easy-to-use instrument for the most exquisite precision of cell biological analyses.

Regarding practicality, CTC isolation needs to be more considered for full automation, easy operation, mass production, and price competitiveness. CCM-CTCD, being fully automated, offers one-click processing and high compatibility to mass production. Of course, the final validation of practicality is a clinical application with real patients. In this report we have demonstrated the successful use of CCM-CTCD in a challenging clinical study of NSCLC, a cancer with high phenotypic CTC plasticity and notoriously low CTC counts using the CellSearch^®^ system 10. We showed that polyphyletic CTCs, indeed, were isolated in intra-patient blood samples, that enumeration using the CCM-CTCD correlates strongly with clinical response to EGFR-TKI therapy as well as clinical status, and that mutational analysis using our isolated CTCs can help guide targeted therapy to overcome acquired drug resistance.

Cell stress is caused by the g-force during centrifugation. Given that the radius that CTCs are located in the BLOOD chamber is 38.1 mm (PBMC layer), the g-force exerted on the CTCs is 266g for 15 min. On the contrary, according to the Ficoll protocol as a representative DGM material, it should be operated with 400 g for 30 min to form the PBMC layer from whole blood. Since our continuous centrifugation approach also allows highly precise extraction of PBMCs at a lower g-force compared to conventional approaches 23, 54, 55, we speculated that the cells after the CTCD process might not be damaged. To confirm the cell intactness, cell viability and proliferation studies were conducted (Figure S3). Results showed that the cell viability after the CTCD isolation process was not statistically significant compared to the original cancer cells and the isolated cancer cells were fairly proliferated, maintaining their morphology similar to the control. Based on the results, the CCM-CTCD platform has the potential to cultivate genuine CTCs exposed to relatively low cell stress conditions during the isolation process.

Insofar as we have overcome key problems holding back CTC diagnosis and have applied these improvements in a challenging clinical setting, we believe this novel CCM-CTCD system will significantly address these unmet needs in clinical oncology and help bring robust CTC diagnosis and monitoring to the clinical stage. Moreover, it should be noted that the generality of this technique offers the possibility, beyond CTC analysis, of yet broader uses in cell biology.

## Conclusion

Collectively, our results indicate that CCM-CTCD captures a broad range of CTC phenotypes and subsequent molecular downstream analysis can contribute valuable information on the molecular biology of CTCs with clinical implications for NSCLC patients. EGFR-mutational profile of CTCs detected by CCM-CTCD correlated well with the profile of circulating tumor DNA (ctDNA). Thus, CTC analysis might become a complementary tool for decision-making in patients where ctDNA is not available in sufficient amounts for mutational analysis. After confirmation of our pilot study in a large-scale multi-center clinical trial, CCM-CTCD based CTC analysis has the potential to be used to optimize targeted therapy in NSCLC. Moreover, CCM-CTCD analysis is not restricted to NSCLC and may therefore open a new avenue for CTC analysis in general.

## Methods

### Experimental design

The objective of this study was to demonstrate full capture of diverse CTC phenotypes (single, clustered, EpCAM(+), EpCAM(-), small-sized, and large-sized CTCs) with the fully automated system and to validate clinical utility in clinical monitoring and subsequent evaluation of therapeutic response in lung cancer patients. To evaluate the CCM-CTCD method in overcoming CTC heterogeneity, the recovery rate was evaluated with EpCAM(+) and EpCAM(-) cancer cells. To model multi-metastatic disease, five cancer cell lines were mixed with different phenotypes (EpCAM(+), EpCAM(-), small-sized, and large-sized CTCs) and the recovery rate was confirmed, emphasizing the isolation performance regardless of CTC heterogeneity. The experiments were repeated more than three times to ensure reproducibility. We enrolled the EGFR mutant lung cancer patients to confirm the isolation of genuine CTCs. EGFR mutations were identified from the isolated CTCs and compared with the mutations from cfDNA and tissue biopsy. 10 mL of peripheral blood were collected from 30 patients who were treated at Yonsei University Hospital Cancer Center on one or more occasions for CCM-CTCD analysis. We performed ddPCR and pyrosequencing to evaluate the EGFR mutation in CTCs obtained from EGFR mutant lung cancer patients. Tumor lesions by CT images were compared with the CTC counts to follow up the status of the cancer patients to validate the therapeutic monitoring with the captured CTCs. For the NSCLC clinical validation study, human blood samples were obtained from Severance Hospital under IRB No. 4-2013-0059. For this study, consent for genetic analysis was also obtained.

### Materials

RPMI 1640, PBS buffer (pH7.4) and Trypsin-EDTA solution were purchased from Welgene (Korea). Dynabead-CD45, fetal bovine serum (FBS) and Anti-Pan Cytokeratin (Alexa Fluor 488) were obtained from Invitrogen (MA, USA). Anti-EpCAM was purchased from Abcam (UK). Antibiotic/Antimycotic solution and Ficoll-paque plus were purchased from GE Healthcare (IL, USA). Formaldehyde solution, Bovine Serum Albumin, Triton X-100, and Hoechst 33342 were acquired from Thermo Scientific (MA, USA). Percoll [pH8.5 - 9.5 (25 °C) cell culture tested] was purchased from Sigma-Aldrich (MO, USA), and Anti-CD45 (Alexa Fluor 594) was obtained from BioLegend (CA, USA). ScreenCell Cyto R kit was purchased from ScreenCell (France) and used according to the instructions given by the manufacturer.

### Disc fabrication

The upper and lower plates of CTCD were machined from polycarbonate slabs (Artryx, Korea) by a CNC milling machine (Figure S4A). The upper plate has valve sockets that are used to contain the ferrowax (Ferrotec, Japan), which forms microvalves to control the flow of samples between chambers in the lower plate. The ferrowax was injected into the sockets with a wax dispenser (Figure S4B). Halbach arrays, which consist of 5 magnets respectively, were inserted into the lower plate (Figure S4C). After that, the upper and lower plates were assembled using double-sided tape patterned by a knife plotter (Figure S4D). Finally, the assembled CTCD was pressed for maximal bonding (3.19 MPa for 60 sec). The disc has a diameter of 140 mm and the size of the largest chamber, the BLOOD chamber, is about 48.7 × 7.0 × 12.0 mm (Figure S5). The BLOOD chamber has a ramp structure that prevents the mixing between the DGM and whole blood. In the middle of each channel, there is more than one valve that prevents liquid transfer between the chambers.

### Cell culture and identification of CTCs

In this study, eight cancer cell lines, lung cancer cells (PC-9, A549, H1975, and H1688), breast cancer cells (MDA-MB-231, SK-BR-3, and MCF-7) and bladder cancer cell (T24) were used as models for CTCs in blood. The cell lines were maintained in RPMI-1640 supplemented with 10% FBS, L-glutamine (300 mg/L), 25 mM HEPES NaHCO_3_, 1% Antibiotic/Antimycotic. All cell lines were cultured at 37 °C in a humidified atmosphere of 5% CO_2_. After the cells reached 80-90% confluence, the medium was removed and replaced with a fresh medium. Prior to each experiment, cells grown to confluence were trypsinized and resuspended in the media as specified.

For CTC recovery assessments, the cells were pre-stained with CellTracker Green CMFDA Dye (Invitrogen), in which labeling was achieved by incubating the cells with the tracking dye (5 μM) for 20 min at 37 °C. The cells were then pelleted by centrifugation (1500 RPM, 3 min), the supernatant was decanted, and the cells were washed twice with PBS to remove any excess dye. Then, the cells were resuspended in media. The concentration of cells was first determined by manual counting using a hemocytometer. Next, the cells were diluted to ideal concentrations of 5-500 cells/100 μL by statistical sampling of a serial dilution. Subsequently, 100 μL of the diluted cell suspension was transferred to a 96-well microplate, using a micropipette and then centrifuged. The number of cells in the transferred suspension was counted under a fluorescence microscope. The cell suspension was spiked to the 0.9 mL of whole blood. After the cell spiking, the remaining cells in the 96-well microplate were counted and this residual number was subtracted from the initially counted cell number, thus deciding the number of spiked cells.

### CTC isolation process

(1) The 0.9 mL of DGM (1.0823 g/mL) was injected into the BLOOD chamber inlet, 0.6 mL of DGM (1.0823 g/mL) was injected into the DEPLETION chamber inlet, 50 μL of Dynabead CD45 was injected into the MIXING chamber, and then centrifuged in the CCM-CTCD device (2000 RPM for 10 sec). (2) Blood samples were injected into the BLOOD chamber inlet (Figure S6). (3) Centrifugation was performed at 150, 300, 375, 450, 600, 750, 900 and 1000 RPM for 70 sec, respectively, and then 2500 RPM for 15 min. (4) Valve was opened by laser irradiation and the plasma was transferred to the PLASMA chamber. (5) Valve was opened, and PBMC with CTCs transferred to the MIXING chamber. (6) Valve was closed to prevent both contamination of blood cells and CTC loss during the mixing process. (7) The disc was shaken (at an angle of 135°, 1 Hz, for 60 min) to bind the microbeads with the WBCs in the MIXING chamber. (8) Valve was opened and the MIXING chamber contents were transferred to the DEPLETION chamber. (9) Centrifugation was performed at 3000 RPM for 5 min to deplete the WBCs. (10) Valve was opened and the contents above the DGM layer were transferred to the CTC chamber. (11) To measure the CTC recovery rate, the sample in this final CTC chamber was transferred into a 96-well microplate by a micropipette (Figure S6). To transfer residual CTCs in the CTC chamber, the CTC chamber was washed by PBS and transferred to the 96-well microplate and the plate was spun at 1000 RPM for 3 min. After extraction of CTC, the used CTCD was discarded. (12) Green fluorescent positive cells were counted as being CTCs. For the residual rates observed in the PLASMA chamber, the following calculations were used, where CTC_initial_ refers to the numbers initial spiked and WBC_initial_ the WBC number as determined by conventional hemocytometry.

Residual Rate_CTC_ = CTC_PLASMA_/CTC_initial_ × 100% (1)

Residual Rate_WBC_ = WBC_PLASMA_/WBC_initial_ × 100% (2)

The CTC recovery rate was calculated as follows, where, again, CTC_initial_ refers to the spiked numbers and for this case CTC_final_ refers to the number of CTCs found in the final, CTC chamber.

CTC Recovery Rate = CTC_final_/CTC_initial_ × 100% (3)

The sample in the CTC chamber was transferred into a 96-well microplate to measure CTC purity. WBCs were stained with Hoechst 33342 (1 mg/mL), centrifuging the plate with 1500 RPM for 3 min. WBCs positively stained for Hoechst were counted by imaging the whole reservoir area. To compute the WBC depletion rate, we used the following equation. The WBC_initial_ was determined by hemocytometry and the WBC_final_ was determined by counting the remaining WBCs in the final CTC chamber.

WBC Depletion Rate = (WBC_initial_ - WBC_final_)/WBC_initial_ × 100% (4)

### Clinical validation

This study was an exploratory trial to evaluate the performance of a newly developed CTC capture system CCM-CTCD for isolating heterogeneous CTCs from cancer patients. We prospectively collected peripheral blood originated from EGFR mutant lung cancer patients. Blood samples in EDTA tubes were immediately transferred and processed with the CCM-CTCD system at room temperature. Medical records and radiologic images of all patients were collected to evaluate demographic and clinic-pathologic parameters, along with tumor response. All enrolled patients underwent follow-up CT scanning to evaluate their clinical responses based on the Response Evaluation Criteria in Solid Tumors (RECIST 1.1). Clinical details of the study participants, as well as EGFR mutation profiles, are provided in Table S2 and S3, respectively.

### Characterization of isolated CTCs

After isolation of CTCs from the CCM-CTCD system, the collected cells were fixed with 4% paraformaldehyde for 1 hour. To identify the types of CTCs, the cells were labeled overnight at 4 °C with a mixture of antibodies, including the anti-PanCK antibody (Alexa Fluor 488), anti-EpCAM antibody and anti-CD45 antibody (Alexa Fluor 594). After washing with PBS three times, the cells were further incubated with DAPI solution (Thermo Scientific) for 10 min and mounted with the Vector shield mounting media (Vector laboratories, CA, USA). CTCs were identified based on the staining [PanCK(+)/CD45(-)/DAPI(+)] and EpCAM expression. The cell size was measured by referring to the previous studies 56. By using the ZEN 3.5 program of the Zeiss microscope (Germany), the size of CTCs was measured by the diameter of cells from bright field images.

### DNA extraction and ddPCR

After collecting blood samples from EGFR mutant patients, plasma was isolated by centrifugation, and the supernatant was collected. Collected plasma was then stored in a cryostat tube at -80 °C. Extraction of cfDNA was then performed using the QIAmp Circulating Nucleic Acid Kit (Qiagen, Germany) according to Qiagen's protocol. Genomic DNA of CTC was isolated using the DNeasy Blood & Tissue Kit (Qiagen) according to the manufacturer's protocol. The ddPCR was performed with Bio-Rad QX200 ddPCR system (Bio-Rad, CA, USA) to validate EGFR Exon 19 deletion (E19del), T790M, or L858R mutation. For the ddPCR of each sample, droplets were generated within a DG8 Cartridge (Bio-Rad) which was preloaded with the sample (20 μL) and the droplet generation oil (70 μL). All droplets were then transferred into a 96-well plate, and sealed with a PX1 PCR Plate Sealer (Bio-Rad). A programmed thermal cycler was set at 96 °C for 10 min, followed by 40 cycles of 94 °C for 30 sec and 60 °C for 60 sec, and finally 98 °C for 10 min. The droplets containing amplicons were quantified with a QX200 Droplet Reader using the QuantaSoft software package (Bio-Rad).

### Statistical analysis

Results in all graphs of recovery and depletion rate for the optimization and performance of CCM-CTC were shown as mean values ± SD with more than three independent experiments and statistically analyzed with an unpaired two-tailed t-test. Asterisks represent statistically significant values (*p < 0.05, **p < 0.01, ***p < 0.001). Correlation between CTC count and tumor T stage was shown as mean values ± SEM and analyzed by an unpaired two-tailed t-test with Welch's correction.

## Supplementary Material

Supplementary methods, figures and tables.

Supplementary movie 1.

Supplementary movie 2.

## Abbreviations

CCM: continuous centrifugal microfluidics
cfDNA: cell-free DNA
CK: cytokeratin
CT: computerized tomography
CTC: circulating tumor cell
CTCD: circulating tumor cell disc
ctDNA: circulating tumor DNA
ddPCR: droplet digital PCR
DGM: density gradient medium
DLD: deterministic lateral displacement
DP: disc part
EGFR: epidermal growth factor receptor
EpCAM: epithelial cell adhesion molecule
FBS: fetal bovine serum
LP: laser part
NSCLC: non-small cell lung cancer
PBMC: peripheral blood mononuclear cells
RBC: red blood cell
SD: standard deviation
TKI: tyrosine kinase inhibitor
WBC: white blood cell

## References

[1] Bourzac K (2014). Biology: Three known unknowns. Nature.

[2] Bocci F, Gearhart-Serna L, Boareto M, Ribeiro M, Ben-Jacob E, Devi GR (2019). Toward understanding cancer stem cell heterogeneity in the tumor microenvironment. Proc Natl Acad Sci USA.

[3] Alix-Panabieres C, Pantel K (2021). Liquid Biopsy: From Discovery to Clinical Application. Cancer Discov.

[4] Alix-Panabieres C, Pantel K (2014). Challenges in circulating tumour cell research. Nat Rev Cancer.

[5] Dawson SJ, Tsui DW, Murtaza M, Biggs H, Rueda OM, Chin SF (2013). Analysis of circulating tumor DNA to monitor metastatic breast cancer. N Engl J Med.

[6] Rossi E, Fabbri F (2019). CTCs 2020: Great Expectations or Unreasonable Dreams. Cells.

[7] Pantel K, Alix-Panabieres C (2019). Liquid biopsy and minimal residual disease - latest advances and implications for cure. Nat Rev Clin Oncol.

[8] Ohnaga T, Takei Y, Nagata T, Shimada Y (2018). Highly efficient capture of cancer cells expressing EGFR by microfluidic methods based on antigen-antibody association. Sci Rep.

[9] Vila A, Abal M, Muinelo-Romay L, Rodriguez-Abreu C, Rivas J, Lopez-Lopez R (2016). EGFR-Based Immunoisolation as a Recovery Target for Low-EpCAM CTC Subpopulation. PLoS One.

[10] de Wit S, van Dalum G, Lenferink ATM, Tibbe AGJ, Hiltermann TJN, Groen HJM (2015). The detection of EpCAM(+) and EpCAM(-) circulating tumor cells. Sci Rep.

[11] Nasiri R, Shamloo A, Ahadian S, Amirifar L, Akbari J, Goudie MJ (2020). Microfluidic-Based Approaches in Targeted Cell/Particle Separation Based on Physical Properties: Fundamentals and Applications. Small.

[12] Chen K, Dopico P, Varillas J, Zhang J, George TJ, Fan ZH (2019). Integration of Lateral Filter Arrays with Immunoaffinity for Circulating-Tumor-Cell Isolation. Angew Chem Int Ed Engl.

[13] Li P, Mao Z, Peng Z, Zhou L, Chen Y, Huang PH (2015). Acoustic separation of circulating tumor cells. Proc Natl Acad Sci USA.

[14] Ozkumur E, Shah AM, Ciciliano JC, Emmink BL, Miyamoto DT, Brachtel E (2013). Inertial focusing for tumor antigen-dependent and -independent sorting of rare circulating tumor cells. Sci Transl Med.

[15] Warkiani ME, Khoo BL, Wu L, Tay AK, Bhagat AA, Han J (2016). Ultra-fast, label-free isolation of circulating tumor cells from blood using spiral microfluidics. Nat Protoc.

[16] Zhao W, Liu Y, Jenkins BD, Cheng R, Harris BN, Zhang W (2019). Tumor antigen-independent and cell size variation-inclusive enrichment of viable circulating tumor cells. Lab Chip.

[17] Hu X, Zhu D, Chen M, Chen K, Liu H, Liu W (2019). Precise and non-invasive circulating tumor cell isolation based on optical force using homologous erythrocyte binding. Lab Chip.

[18] Mishra A, Dubash TD, Edd JF, Jewett MK, Garre SG, Karabacak NM (2020). Ultrahigh-throughput magnetic sorting of large blood volumes for epitope-agnostic isolation of circulating tumor cells. Proc Natl Acad Sci USA.

[19] Kim MS, Kim J, Lee W, Cho SJ, Oh JM, Lee JY (2013). A trachea-inspired bifurcated microfilter capturing viable circulating tumor cells via altered biophysical properties as measured by atomic force microscopy. Small.

[20] Campton DE, Ramirez AB, Nordberg JJ, Drovetto N, Clein AC, Varshavskaya P (2015). High-recovery visual identification and single-cell retrieval of circulating tumor cells for genomic analysis using a dual-technology platform integrated with automated immunofluorescence staining. BMC Cancer.

[21] Rosenberg R, Gertler R, Friederichs J, Fuehrer K, Dahm M, Phelps R (2002). Comparison of two density gradient centrifugation systems for the enrichment of disseminated tumor cells in blood. Cytometry.

[22] Nel I, Gauler TC, Bublitz K, Lazaridis L, Goergens A, Giebel B (2016). Circulating Tumor Cell Composition in Renal Cell Carcinoma. PLoS One.

[23] Cui C, Schoenfelt KQ, Becker KM, Becker L (2021). Isolation of polymorphonuclear neutrophils and monocytes from a single sample of human peripheral blood. STAR protocols.

[24] Drucker A, Teh EM, Kostyleva R, Rayson D, Douglas S, Pinto DM (2020). Comparative performance of different methods for circulating tumor cell enrichment in metastatic breast cancer patients. PLoS One.

[25] Ramirez AB, U'Ren L, Campton DE, Stewart D, Nordberg JJ, Stilwell JL (2017). RareCyte(R) CTC Analysis Step 1: AccuCyte(R) Sample Preparation for the Comprehensive Recovery of Nucleated Cells from Whole Blood. Methods Mol Biol.

[26] Maertens Y, Humberg V, Erlmeier F, Steffens S, Steinestel J, Bogemann M (2017). Comparison of isolation platforms for detection of circulating renal cell carcinoma cells. Oncotarget.

[27] Banko P, Lee SY, Nagygyorgy V, Zrinyi M, Chae CH, Cho DH (2019). Technologies for circulating tumor cell separation from whole blood. J Hematol Oncol.

[28] Rushton AJ, Nteliopoulos G, Shaw JA, Coombes RC (2021). A Review of Circulating Tumour Cell Enrichment Technologies. Cancers (Basel).

[29] Kallergi G, Politaki E, Alkahtani S, Stournaras C, Georgoulias V (2016). Evaluation of Isolation Methods for Circulating Tumor Cells (CTCs). Cell Physiol Biochem.

[30] Sharma S, Zhuang R, Long M, Pavlovic M, Kang Y, Ilyas A (2018). Circulating tumor cell isolation, culture, and downstream molecular analysis. Biotechnol Adv.

[31] Park JM, Kim MS, Moon HS, Yoo CE, Park D, Kim YJ (2014). Fully Automated Circulating Tumor Cell Isolation Platform with Large-Volume Capacity Based on Lab-on-a-Disc. Anal Chem.

[32] Gertler R, Rosenberg R, Fuehrer K, Dahm M, Nekarda H, Siewert JR (2003). Detection of circulating tumor cells in blood using an optimized density gradient centrifugation. Recent Results Cancer Res.

[33] Kowalik A, Kowalewska M, Gozdz S (2017). Current approaches for avoiding the limitations of circulating tumor cells detection methods-implications for diagnosis and treatment of patients with solid tumors. Transl Res.

[34] Kim MS, Sim TS, Kim YJ, Kim SS, Jeong H, Park JM (2012). SSA-MOA: a novel CTC isolation platform using selective size amplification (SSA) and a multi-obstacle architecture (MOA) filter. Lab Chip.

[35] Lee SJ, Sim TS, Shin HY, Lee J, Kim MY, Sunoo J (2019). Microslit on a chip: A simplified filter to capture circulating tumor cells enlarged with microbeads. PLoS One.

[36] Punnoose EA, Atwal S, Liu W, Raja R, Fine BM, Hughes BG (2012). Evaluation of circulating tumor cells and circulating tumor DNA in non-small cell lung cancer: association with clinical endpoints in a phase II clinical trial of pertuzumab and erlotinib. Clin Cancer Res.

[37] Krebs MG, Hou JM, Sloane R, Lancashire L, Priest L, Nonaka D (2012). Analysis of circulating tumor cells in patients with non-small cell lung cancer using epithelial marker-dependent and -independent approaches. J Thorac Oncol.

[38] Hosokawa M, Kenmotsu H, Koh Y, Yoshino T, Yoshikawa T, Naito T (2013). Size-based isolation of circulating tumor cells in lung cancer patients using a microcavity array system. PLoS One.

[39] Fachin F, Spuhler P, Martel-Foley JM, Edd JF, Barber TA, Walsh J (2017). Monolithic Chip for High-throughput Blood Cell Depletion to Sort Rare Circulating Tumor Cells. Sci Rep.

[40] Russano M, Napolitano A, Ribelli G, Iuliani M, Simonetti S, Citarella F (2020). Liquid biopsy and tumor heterogeneity in metastatic solid tumors: the potentiality of blood samples. J Exp Clin Cancer Res.

[41] Nagrath S, Sequist LV, Maheswaran S, Bell DW, Irimia D, Ulkus L (2007). Isolation of rare circulating tumour cells in cancer patients by microchip technology. Nature.

[42] Vermesh O, Aalipour A, Ge TJ, Saenz Y, Guo Y, Alam IS (2018). An intravascular magnetic wire for the high-throughput retrieval of circulating tumour cells *in vivo*. Nat Biomed Eng.

[43] Chen P, Wang Y, He Y, Huang K, Wang X, Zhou R (2021). Homogeneous Visual and Fluorescence Detection of Circulating Tumor Cells in Clinical Samples via Selective Recognition Reaction and Enzyme-Free Amplification. ACS Nano.

[44] Kang H, Kim J, Cho H, Han KH (2019). Evaluation of Positive and Negative Methods for Isolation of Circulating Tumor Cells by Lateral Magnetophoresis. Micromachines (Basel).

[45] Gribko A, Stiefel J, Liebetanz L, Nagel SM, Kunzel J, Wandrey M (2021). IsoMAG-An Automated System for the Immunomagnetic Isolation of Squamous Cell Carcinoma-Derived Circulating Tumor Cells. Diagnostics (Basel).

[46] Jou HJ, Chou LY, Chang WC, Ho HC, Zhang WT, Ling PY (2021). An Automatic Platform Based on Nanostructured Microfluidic Chip for Isolating and Identification of Circulating Tumor Cells. Micromachines (Basel).

[47] Kim J, Cho H, Kim J, Park JS, Han KH (2021). A disposable smart microfluidic platform integrated with on-chip flow sensors. Biosens Bioelectron.

[48] Wang J, Li Y, Wang R, Han C, Xu S, You T (2021). A Fully Automated and Integrated Microfluidic System for Efficient CTC Detection and Its Application in Hepatocellular Carcinoma Screening and Prognosis. ACS Appl Mater Interfaces.

[49] Liu Z, Huang Y, Liang W, Bai J, Feng H, Fang Z (2021). Cascaded filter deterministic lateral displacement microchips for isolation and molecular analysis of circulating tumor cells and fusion cells. Lab Chip.

[50] Abdulla A, Zhang T, Li S, Guo W, Warden AR, Xin Y (2022). Integrated microfluidic single-cell immunoblotting chip enables high-throughput isolation, enrichment and direct protein analysis of circulating tumor cells. Microsyst Nanoeng.

[51] Zhang X, Wei X, Men X, Wu CX, Bai JJ, Li WT (2021). Dual-Multivalent-Aptamer-Conjugated Nanoprobes for Superefficient Discerning of Single Circulating Tumor Cells in a Microfluidic Chip with Inductively Coupled Plasma Mass Spectrometry Detection. ACS applied materials & interfaces.

[52] Zeinali M, Lee M, Nadhan A, Mathur A, Hedman C, Lin E (2020). High-Throughput Label-Free Isolation of Heterogeneous Circulating Tumor Cells and CTC Clusters from Non-Small-Cell Lung Cancer Patients. Cancers (Basel).

[53] Petchakup C, Tay HM, Li KHH, Hou HW (2019). Integrated inertial-impedance cytometry for rapid label-free leukocyte isolation and profiling of neutrophil extracellular traps (NETs). Lab Chip.

[54] Weller P, Nel I, Hassenkamp P, Gauler T, Schlueter A, Lang S (2014). Detection of circulating tumor cell subpopulations in patients with head and neck squamous cell carcinoma (HNSCC). PLoS One.

[55] Kong BS, Lee C, Cho YM (2022). Protocol for the assessment of human T cell activation by real-time metabolic flux analysis. STAR protocols.

[56] Lee HJ, Oh JH, Oh JM, Park JM, Lee JG, Kim MS (2013). Efficient isolation and accurate *in situ* analysis of circulating tumor cells using detachable beads and a high-pore-density filter. Angew Chem Int Ed Engl.

