# Supplementary material for: Continuous centrifugal microfluidics (CCM) isolates heterogeneous circulating tumor cells via full automation
Source: Theranostics. 2022 May 1;12(8):3676–89. doi: 10.7150/thno.72511 (PMC9131262; doi:10.7150/thno.72511)
Supplement: Supplementary file 1 — Supplementary methods, figures and tables. [file thnov12p3676s1.pdf]

Supporting Information

Continuous centrifugal microfluidics (CCM) isolates heterogeneous circulating tumor cells via full automation

Hyeong Jung Woo[1#], Seung-Hoon Kim[2#], Hyo Jung Kang[2#], Soo-Hwan Lee[3#], Seung Joon Lee[2], Jong Man Kim[2], Ogan Gurel[2,4], Soo Yeol Kim[1], Hye Ran Roh[2], Jungmin Lee[2], Yeonsoo Park[1], Hyun Young Shin[2], Yong-Il Shin[5,6], Sun Min Lee[6,7], So Yeon Oh[8], Young Zoon Kim[9], Jung-Il Chae[10], Seoyoung Lee[11], Min Hee Hong[12], Byoung Chul Cho[12], Eun Sook Lee[13], Klaus Pantel[14], Hye Ryun Kim[12]* and Minseok S. Kim[1,2]*

1. Department of New Biology, DGIST, Daegu 42988, Korea

2. CTCELLS Inc., 206-C, R7, DGIST, Daegu 42988, Korea

3. JE-UK Institute for Cancer Research, JEUK Co. Ltd., Gumi-si 39418, Korea

4. College of Transdisciplinary Studies, DGIST, Daegu 42988, Korea

5. Research Institute for Convergence of Biomedical Science and Technology, Pusan National University Yangsan Hospital, Yangsan 50612, Korea

6. Department of Rehabilitation Medicine, Pusan National University School of Medicine, Yangsan 50612, Korea

7. Department of Laboratory Medicine, Pusan National University Yangsan Hospital, Pusan National University School of Medicine, Yangsan 50612, Korea

8. Medical Oncology & Hematology, Department of Internal Medicine, Pusan National University Yangsan Hospital, Yangsan 50612, Korea

9. Division of Neurooncology and Department of Neurosurgery, Samsung Changwon Hospital, Sungkyunkwan University School of Medicine, Changwon 51353, Korea

10. Department of Dental Pharmacology, School of Dentistry, Jeonbuk National University, Jeonju 54896, Korea.

11. Division of Medical Oncology, Department of Internal Medicine, Yonsei University College of Medicine, Gangnam Severance Hospital, Seoul 06273, Korea

12. Division of Medical Oncology, Department of Internal Medicine, Yonsei Cancer Center, Yonsei University College of Medicine, Seoul 03722, Korea

13. Center for Breast Cancer, National Cancer Center, Goyang 10408, Korea

14. Institute of Tumour Biology, University Medical Centre Hamburg-Eppendorf, Hamburg 20246, Germany

#These authors equally contributed to this work.

*Corresponding authors: Hye Ryun Kim, Division of Medical Oncology, Department of Internal Medicine, Yonsei Cancer Center, Yonsei University College of Medicine, Seoul 03722, Korea. E-mail: NOBELG@yuhs.ac. Minseok S. Kim, Department of New Biology, DGIST, Daegu 42988, Korea. E-mail: kms@dgist.ac.kr

The supporting information includes:

- Supplementary Methods
- Supplementary Figures
- Supplementary Tables
- Supplementary Movies

Supplementary Methods

Evaluation of cell intactness after the CTCD process

To examine the cell damage caused by CCM-CTCD isolation, we compared the viability and proliferation ability of CCM-CTCD isolated cancer cells and control cells. After the isolation process, the viability of cancer cells was analyzed by propidium iodide (PI) staining. PC-9 cancer cells stained with CellTracker Green were spiked in the whole blood. During the CCM-CTCD processing, the control cells were stored at room temperature. After the operation of the CCM-CTCD, the cancer cells were collected from the disc. The collected cells and the control were stained with PI and analyzed by a fluorescent microscope. The total cell number is calculated as green positive, and the number of dead cells is calculated as green and red double positive. The viability of cancer cells was calculated by dividing the difference between the number of green and double-positive cells by the number of green positive cells. To evaluate the proliferation ability after the CCM-CTCD isolation process, the isolated PC-9 cells were cultured for 7 days in the 48 well plates.

Supplementary Figures

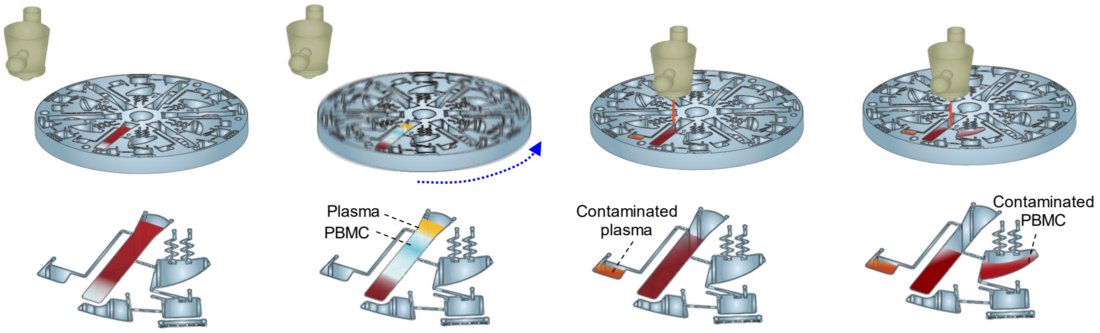

Figure S1. Conventional laser irradiated centrifugal microfluidic system. After separating the blood layer, the disc must be stopped for the laser to operate, resulting in spreading the fluid layers. This collapse of the separation results in the imprecise extraction of fluids through the microfluidic channels.

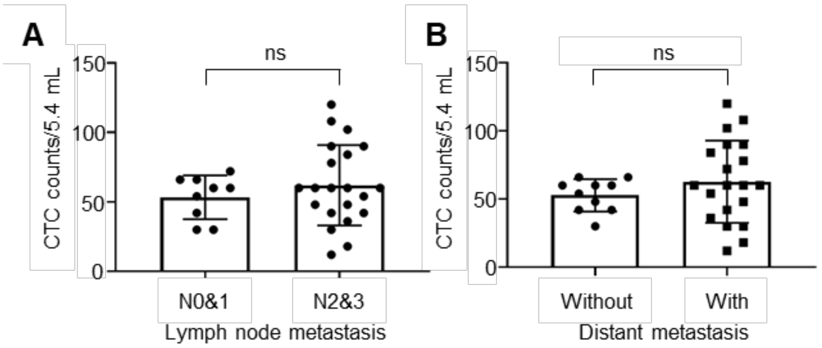

Figure S2. Relationship between CTC counts and clinical relevance. Correlation between CTC counts with **(A)** regional lymph nodes metastasis and **(B)** distant metastasis. Depending on the N (regional lymph nodes) or the M (distant metastasis) stage, there was no obvious difference between CTC counts and the patients' stage.

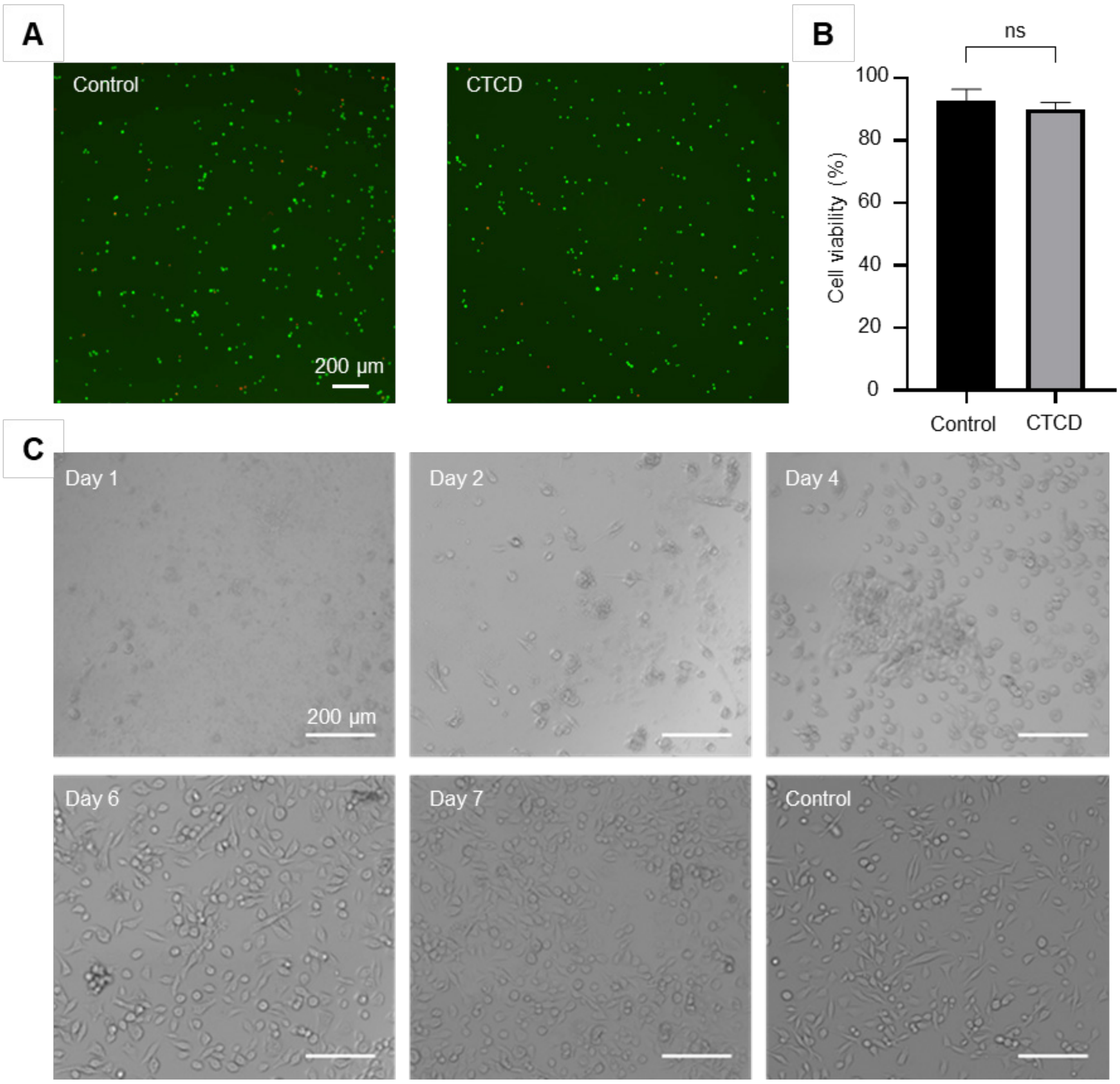

Figure S3. Viability of cancer cells after isolation. (A) Fluorescent image of propidium iodide-stained control cancer cells and CCM-CTCD isolated cancer cells. **(B)** Viability comparison of CCM-CTCD isolated cancer cells and control. CCM-CTCD isolated cells and control cells did not show significant differences in viability. **(C)** Culture of the isolated cancer cell. The proliferation of cancer cells is maintained after the isolation process.

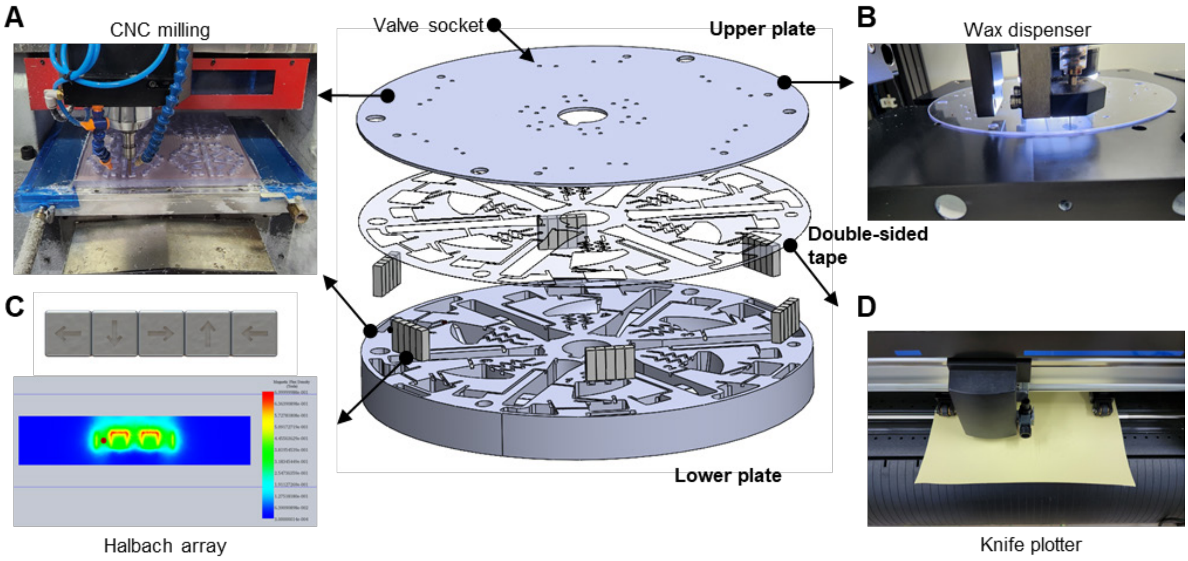

Figure S4. CTCD fabrication process. (A) The upper and lower plates of CTCD were machined from polycarbonate slabs by a CNC milling machine. **(B)** The upper plate has valve sockets to contain ferrowax, which is injected with a precise volume-controlled wax dispenser. **(C)** Halbach arrays to maximize magnetic force are inserted into the lower plate. **(D)** The upper and lower plates are assembled by double-sided tape patterned by a knife plotter.

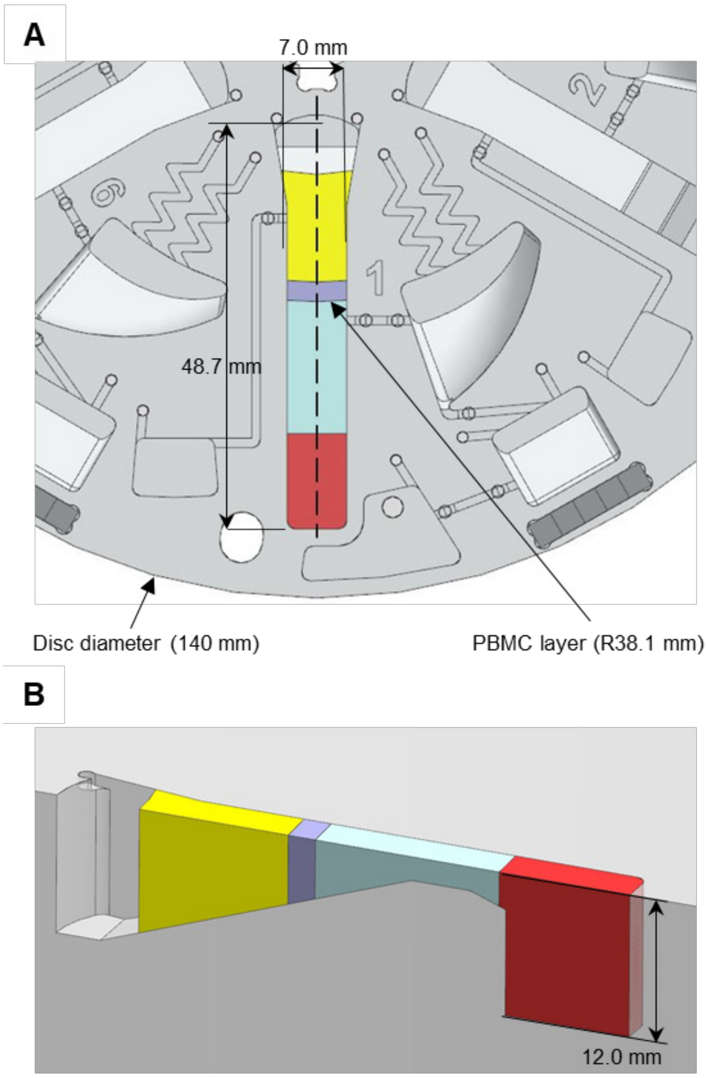

Figure S5. Dimension of CTCD. (A) Detailed dimension of the CTCD. The colors yellow, blue, sky-blue, and red indicate plasma, PBMC, DGM, and RBC layers, respectively. R indicates the radius from the center of the disc. **(B)** The cross-section view of the BLOOD chamber. The BLOOD chamber has a ramp to prevent the mixing between whole blood and DGM.

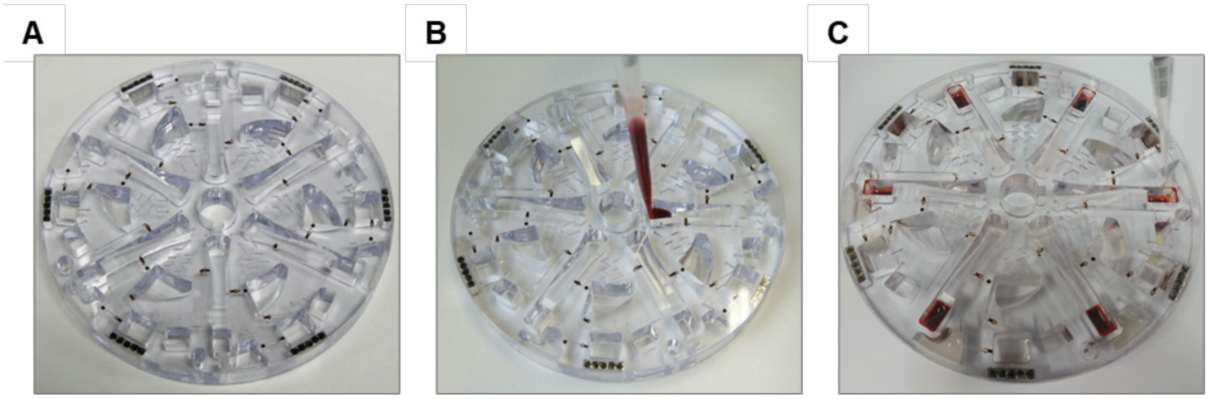

Figure S6. Blood injection and CTC collection. (A) An image of the CTCD. **(B)** Injection of whole blood into the BLOOD chamber via the inlet port of the CTCD. **(C)** Collection of the CTCs from the CTC chamber via micropipette.

Supplementary Tables

Table S1. Isolation sequence and turn-around time for CCM-CTCD for individual action. Laser operation and rotor-operated centrifugation conditions include rotating, acceleration, duration, and deceleration of CTCD in the CCM-CTCD system.

	Performance	Laser	Rotor operation				Time (sec)
			Spin	Acceleration (sec)	Duration (sec)	Deceleration (sec)	
BLOOD chamber	Centrifugation	-	150	10	60	-	70
	Centrifugation	-	300	10	60	-	70
	Centrifugation	-	375	10	60	-	70
	Centrifugation	-	450	10	60	-	70
	Centrifugation	-	600	10	60	-	70
	Centrifugation	-	750	10	60	-	70
	Centrifugation	-	900	10	60	-	70
	Centrifugation	-	1000	10	60	-	70
	Centrifugation	-	2500	30	900	100	1030
	Valve 1 open	5W, 5s	-	-	-	-	5
	Centrifugation	-	1000	5	20	10	35
	Valve 2 open	5W, 5s	-	-	-	-	5
	Centrifugation	-	300	-	60	-	60
	Centrifugation		2000	5	20	10	35
	Valve 3 close	5W, 5s	-	-	-	-	-
MIXING chamber	Shaking	-	135°, 1Hz	-	3600	-	3600
	Valve 4 open	5W, 5s	-	-	-	-	5
	Centrifugation	-	300	-	60	-	60
DEPLETION chamber	Centrifugation	-	3000	60	300	120	480
	Valve 5 open	5W, 5s	-	-	-	-	5

	Centrifugation	-	300	-	60	-	60
	Centrifugation		2000	5	20	10	35
Total time	~ 100 min						5975

Table S2. Summary of EGFR mutation profiles of the 30 study subjects using ddPCR and pyrosequencing.

No.	ddPCR		Pyrosequencing	
	Blood	CTC	Blood	CTC
1	L858R	L858R	L858R	L858R
2	E19Del/T790M	E19Del/T790M	T790M	T790M
3	E19Del/T790M	E19Del/T790M	T790M	T790M
4	E19Del	E19Del	-	-
5	E19Del/T790M	E19Del/T790M	T790M	T790M
6	E19Del/T790M	E19Del/T790M	T790M	T790M
7	E19Del/T790M	E19Del/T790M	T790M	T790M
8	E19Del/T790M	E19Del/T790M	T790M	T790M
9	E19Del/T790M	E19Del/T790M	T790M	T790M
10	T790M	T790M	T790M	T790M
11	E19Del/T790M	E19Del/T790M	T790M	T790M
12	E19Del/T790M	E19Del/T790M	T790M	T790M
13	E19Del/T790M	E19Del/T790M	T790M	T790M
14	E19Del/T790M	E19Del/T790M	T790M	T790M
15	E19Del/T790M	E19Del/T790M	T790M	T790M
16	E19Del/T790M	E19Del/T790M	T790M	T790M
17	E19Del/T790M	E19Del/T790M	T790M	T790M
18	E19Del/T790M	E19Del/T790M	T790M	T790M
19	L858R/T790M	L858R/T790M	L858R/T790M	L858R/T790M
20	E19Del/T790M	E19Del/T790M	T790M	T790M
21	E19Del/T790M	E19Del/T790M	T790M	T790M
22	L858R/T790M	L858R/T790M	L858R/T790M	L858R/T790M
23	E19Del/T790M	E19Del/T790M	T790M	T790M
24	E19Del/T790M	E19Del/T790M	T790M	T790M
25	L858R/T790M	L858R/T790M	L858R/T790M	L858R/T790M
26	L858R/T790M	L858R/T790M	L858R/T790M	L858R/T790M
27	E19Del/T790M	E19Del/T790M	T790M	T790M
28	E19Del/T790M	E19Del/T790M	T790M	T790M
29	E19Del/T790M	E19Del/T790M	T790M	T790M
30	L858R/T790M	L858R/T790M	L858R/T790M	L858R/T790M

Table S3. Clinical characteristics of the 30 study subjects.

No.	Age/Sex	Smoking	Stage	Distant metastasis	CTC counts/5.4mL	CTC cluster detected	EGFR mutation			Previous EGFR TKI therapy
							Tissue	cfDNA	CTC	
1	M/67	2	IV	Y	78	N	L858R	L858R	L858R	None
2	F/48	0	IV	Y	12	Y	E19Del	E19Del T790M	E19Del T790M	None
3	M/55	2	IV	Y	36	N	E19Del	E19Del T790M	E19Del T790M	None
4	F/68	0	IV	N	60	N	E19Del	E19Del	E19Del	None
5	F/69	0	IV	Y	120	Y	E19Del T790M	E19Del T790M	E19Del T790M	G
6	M/74	0	IV	Y	60	N	E19Del T790M	E19Del T790M	E19Del T790M	G
7	F/55	0	IV	Y	72	N	E19Del T790M	E19Del T790M	E19Del T790M	E
8	F/71	0	IV	Y	60	N	E19Del T790M	E19Del T790M	E19Del T790M	A, E
9	M/63	2	IV	N	48	N	E19Del T790M	E19Del T790M	E19Del T790M	G, A, E
10	F/65	0	IV	N	60	N	L858R**	T790M	T790M	E
11	F/73	0	IV	Y	42	N	E19Del T790M	E19Del T790M	E19Del T790M	E
12	F/62	0	IV	Y	90	N	E19Del*	E19Del T790M	E19Del T790M	G
13	F/60	0	IV	Y	108	N	E19Del	E19Del T790M	E19Del T790M	G
14	M/43	0	III	Y	30	N	E19Del	E19Del T790M	E19Del T790M	A
15	F/41	0	IV	N	30	N	E19Del	E19Del T790M	E19Del T790M	G
16	F/72	0	IV	N	42	N	E19Del	E19Del T790M	E19Del T790M	G
17	M/64	2	III	Y	102	N	E19Del***	E19Del T790M	E19Del T790M	G, A
18	M/69	0	IV	Y	18	N	E19Del T790M	E19Del T790M	E19Del T790M	A
19	F/72	0	III	N	54	N	L858R***	L858R T790M	L858R T790M	G
20	F/58	1	IV	Y	48	N	E19Del T790M***	E19Del T790M	E19Del T790M	G, O
21	M/72	2	IV	Y	54	N	E19Del***	E19Del T790M	E19Del T790M	G, E, O
22	M/62	1	IV	Y	90	N	L858R T790M	L858R T790M	L858R T790M	G, O
23	F/28	0	IV	Y	30	N	E19Del	E19Del T790M	E19Del T790M	O
24	F/78	0	IV	Y	84	N	E19Del T790M	E19Del T790M	E19Del T790M	G, O
25	F/61	0	IV	Y	60	N	L858R**	L858R T790M	L858R T790M	G, O
26	F/72	0	IV	N	66	N	L858R	L858R T790M	L858R T790M	O
27	F/62	0	IV	Y	60	N	E19Del**	E19Del T790M	E19Del T790M	A, O
28	M/70	2	IV	N	42	N	E19Del*	E19Del T790M	E19Del T790M	O
29	F/57	2	IV	N	60	N	E19Del**	E19Del T790M	E19Del T790M	G, E, O
30	M/61	1	IV	N	66	N	L858R*	L858R T790M	L858R T790M	A, E, O

Smoking: Never Smoker = 0, Former Smoker = 1, Current Smoker = 2

EGFR-TKI: Erlotinib, E; Gefitinib, G; Afatinib, A; Osimertinib, O

* Tumor tissue EGFR mutation detected 1 year ago
**Tumor tissue EGFR mutation detected 2 years ago
***Tumor tissue EGFR mutation detected 3 years ago
